# Interaction of Socioeconomic Status with Risky Internet Use, Gambling and Substance Use in Adolescents from a Structurally Disadvantaged Region in Central Europe

**DOI:** 10.3390/ijerph17134803

**Published:** 2020-07-03

**Authors:** Benjamin Petruzelka, Jaroslav Vacek, Beata Gavurova, Matus Kubak, Roman Gabrhelik, Vladimir Rogalewicz, Miroslav Bartak

**Affiliations:** 1 Department of Addictology, General University Hospital in Prague, Apolinářská 447/4, 128 00 Prague, Czech Republic; benjamin.petruzelka@lf1.cuni.cz (B.P.); jaroslav.vacek@lf1.cuni.cz (J.V.); roman.gabrhelik@lf1.cuni.cz (R.G.); vladimir.rogalewicz@lf1.cuni.cz (V.R.); miroslav.bartak@lf1.cuni.cz (M.B.); 2 Department of Addictology, Charles University, First Faculty of Medicine, Apolinářská 447/4, 128 00 Prague, Czech Republic; 3 Faculty of Economics, Technical University of Košice, 04001 Košice, Slovak Republic; matus.kubak@tuke.sk; 4 Department of Social work, J.E. Purkyně University in Ústí nad Labem, Moskevská 54, 400 96 Ústí nad Labem, Czech Republic

**Keywords:** substance use, gambling, internet use, students, adolescents, socioeconomic status, disadvantaged region

## Abstract

Background and aims: The current level of knowledge concerning the effect of socioeconomic status (SES) on internet use, gambling, and substance use in structurally disadvantaged regions is scarce. The objective of this study was an investigation of the relationship between SES and risky internet use, gambling and substance use in a structurally disadvantaged region in Central Europe. Methods: A cross-sectional survey was conducted among high school students (*n* = 1063) in a Czech structurally disadvantaged region in autumn 2017. Binary Logistic Regression models were applied to data from the modified Excessive Internet Use scale (mEIUS), a standard tool for measuring the risk of addictive behavior on the internet and the risk of excessive gaming. Other data were collected using the Lie/Bet (problematic gambling), CAGE (acronym of the key words: cut, angry, guilty and eye-opener), and the Cannabis Abuse Screening Test (CAST) (problematic alcohol/cannabis use) tools. Results: There were statistically significant differences between at-risk and not-at-risk groups in addictive behavior on the internet and gaming, while none were found in problematic gambling. Individual dimensions of SES showed significant effects on substance use. Regarding parenting styles, significant differences were found only in the risk of addictive behavior on the internet or gaming between the authoritarian and authoritative styles. Being engaged in behavioral addictions with one´s parents increased the odds of the behavioral addiction risk and decreased the odds of the substance addiction risk. Engagement with one´s parents in substance addictions decreased the odds of the behavioral addiction risk and increased the odds of the substance addiction risk. Discussion and Conclusions: The results point at specific relations between SES and the risk of addictive behaviors on the internet and gaming within structurally disadvantaged regions. The results of SES and/or structurally disadvantaged region measures obtained in research, policy-making, and care-provision may improve the focus of actions taken.

## 1. Introduction

Socially disadvantaged youth is one of the social groups that are considered vulnerable. When developing appropriate health and social programs and measures, it is important to take into account specific factors linked to youth in structurally disadvantaged regions or neighborhoods [1]. Structurally disadvantaged regions are characterized by lower development in socio-economic indicators such as economic development and unemployment [2]. Differences between regions within European countries are growing [3], and the issue of structurally disadvantaged regions becomes urgent. At a European level, one objective of the drug strategy is to improve preventive measures, taking into account cultural and social factors [4]. Therefore, a study of the influence of socioeconomic status (SES) on gambling, internet use, and/or substance use in adolescents from a structurally disadvantaged region may provide useful insights.

Regarding SES and both gambling and internet use, Lee and McKenzie [5] highlighted the lack of research focused on risky behaviors connected with the use of the internet, and Reith [6] accented the same concerning gambling. As for gambling, Molinaro et al. [7] did not find any relationship between parental education, family structure, and gambling. Consistent with Vitaro et al. [8] and Langhinrichsen-Rohling et al. [9], the analysis by Molinaro et al. [7] showed that family structural characteristics do not have an influence on gambling compared to family relationship characteristics (family support, awareness of free time activities, and rules). However, in a more recent study, Molinaro et al. [10] described the effect of family economic status, the parental education, and the structure of the family on gambling prevalence. The families with medium economic status had significantly lower odds of gambling, while stepfamilies and other family structures except single-parent families had significantly higher odds of gambling than traditional families [10]. The parental education had a rather small effect; the father´s high schooling increased the odds of gambling and the mother´s decreased the odds of gambling [10]. Furthermore, in a recent systematic review, Henkel and Zemlin [11] reported on the relationship between SES and problematic gambling, when the unemployment and migration status were associated with higher prevalence of problematic gambling. The review of longitudinal studies by Dowling et al. [12] also revealed the effect of socioeconomic status on the addiction in question, and Canale et al. [13] examined the influence of socioeconomic status on problematic gaming, both at the individual and regional levels, showing that students living in unequal and poorer regions have higher odds of at-risk gambling. Elgar´s study [14] suggested that youth who live in economically unequal settings and perceive a lack of social support are at higher risk of disordered gambling. The relationship between gambling and SES might be also explained by the higher density of electronic gambling machines in areas with lower SES [15]. Based on a longitudinal study, Auger et al. [16] showed that individuals with lower SES and impulsivity had earlier age of gambling onset. Van der Maas [17] showed—although among adults—that social setting, which has been downplayed in current problem gambling research, has an important influence on gambling.

In the literature, diverse results were found regarding SES and risky internet use. Blinka et al. [18] did not find any influence of the family type and parental education on an excessive use of the internet. Additionally, Spilková et al. [19] found no connection between the family environment and an excessive internet use. On the contrary, Durkee et al. [20] found that the composition of the parents’ household and their employment had an impact on problematic internet use, parental unemployment, and absence of biological parents increasing the risk of problematic use. Lee and McKenzie [5] also found a relationship between low SES and internet addiction, and Ko et al. [21] found that single-parent families are at a higher risk. More recent results from Slovakia showed evidence of the relationship between SES and excessive internet use [22,23]. Urbanová [22] found that adolescents with an unemployed father and perceived low family wealth were more at-risk of excessive internet use. Another study showed that overprotection and lower socioeconomic status were related to the risk of excessive internet use. Contradictory findings were also reported regarding SES and substance use among adolescents [14,15,16,17,18,19,20,21,22,23,24,25,26]. The review by Patrick et al. [27] found evidence of a relationship between SES and substance use, although the shape of this relationship varied.

One explanation for the different findings regarding the role of SES in all three types of risky behaviors discussed in this paper, besides differences in research methodology used in the studies, may be that the social structure and its effect on risky behaviors vary across countries with different social structures. For example, this might have been the case of Molinaro et al. [7], who did not take into account the different structures of countries included in the model. Similarly, the study on internet use by Blinka et al. [18] used a single model for all different countries. Studies that took SES into consideration have been listed as well, e.g., one study of repeated drunkenness showed that the relationship between SES and repeated drunkenness differed between countries [28]. Canale et al. [13] and Gori et al. [29] identified regional differences in the problematic gaming incidence within Italy that corresponded to the socioeconomic characteristics of the regions. Exploration of the connection between substance or behavioral addictions and the socioeconomic inequality at country levels were recommended for the objective of further studies, according to Molinaro et al. [10] and Reith [6] in the case of youth gambling, and according to Galea et al. [30] in the case of substance use.

Our study is focused on the relationship between addictive behaviors (internet use, gaming, and gambling), substance use, and SES in a structurally disadvantaged region. In contrast to previous studies focusing on the national-wide [19] or all-European [10,18] populations of adolescents, we focused on adolescents in a structurally disadvantaged region. According to our knowledge, this is the first study to investigate the relationship between SES and the risk of behavioral addictions in a structurally disadvantaged region among the student population. The aim of this was to study the relationship between SES and risky internet use, gambling, and substance use in structurally disadvantaged region particularly in the Ústí nad Labem Region, one of the most structurally disadvantaged regions in Central Europe. In many terms, the Ústí nad Labem Region may be considered an example of a structurally disadvantaged region in Central Europe, including lower life expectancy, higher poverty rate, high unemployment, poor educational structure, negative internal migration from the region, and a high number of socially excluded locations (see [31]).

## 2. Methods

The research was carried out as a cross-sectional survey among high school students in the Ústí nad Labem Region in the Czech Republic in autumn 2017.

### 2.1. Setting

We focused on a specific region of the Czech Republic that can be referred to as socially disadvantaged or deprived. The Ústí nad Labem Region represents a combination of problematic social conditions, high prevalence of drug use, riskier demographic development, lower health status, and lower levels of education and religiosity [31,32,33]. This region allowed us to monitor the interaction of SES with the rates of risky internet use, gambling, and substance use.

### 2.2. Sample

Out of 53 secondary schools established by the regional administration, 17 schools (upper secondary vocational schools and upper secondary general schools, with school codes 353—vocational training schools; 354—secondary schools with graduation exam and 343—grammar schools according to the International Standard Classification of Education (ISCED) [34]) representing 1063 students aged 15–19 years agreed to participate in the research. The participation of schools was voluntary and unpaid.

### 2.3. Data Collection

Data were collected by trained members of the research team during regular 45-min teaching hours. The majority of the data (590 questionnaires, 56%) was pen-paper collected, while a minority of the data (473 questionnaires, 44%) were collected via computer. Both modes of data collection used similar questionnaires and the data are comparable. The mode of the collection was based on the preferences agreed with individual schools taking part in the survey. The questionnaire consisted of SES assessment and risky behavior measures—excessive internet use and excessive gaming, problematic gambling, daily cigarette smoking, alcohol and cannabis use, parenting styles, and questions about risky activities with parents. Altogether, 1047 valid questionnaires were collected and included in the statistical analysis; only 16 questionnaires were excluded (due to a declaration of use of a non-existent drug “Semeron,” and/or repeated discrepancies in reported prevalence, e.g., the respondent repeatedly reported a more frequent use in the last month than in the whole lifetime).

### 2.4. Measures

The Modified Excessive Internet Use scale (mEIUS) [18] was used to measure the risk of addictive behavior on the internet and the risk of excessive gaming. The Lie/Bet Questionnaire [35] was used to measure the risk of problematic gambling, and the CAGE questionnaire [36] and the Cannabis Abuse Screening Test (CAST) [37] were used to measure the risk of problematic use of alcohol and cannabis respectively. Finally, the standard European School Survey Project on Alcohol and Other Drugs (ESPAD) question was used to measure daily smoking in the last month. The variables were dichotomized, acquiring two levels of problematic behavior, at-risk and not-at-risk. The result was classified as at-risk in the following cases: mEIUS > 2.5, Lie/Bet > 0, CAGE > 0, CAST > 2, and one or more cigarettes smoked daily in the last 30 days.

SES was measured by variables that were divided into three dimensions: education-based SES (educational level of parents according to ESPAD), subjective assessment of SES (family financial status according to ESPAD, students´ own financial resources), and stability of the family background (frequency of moving and family composition). The following variables were recorded to control for the influence of SES: the demographic characteristics of respondents and data concerning their study (sex, year of study and type of school, repeating of a school year (class)—yes or no, expulsion from school—yes or no), a characteristic representing the parenting style (24 questions were used to construct a single coded variable characterizing one of four parenting styles (authoritative, authoritarian, indulgent, and neglectful)) based on an evaluation of the control and warmness [38], and a joint participation of children and parents in risky activities.

### 2.5. Statistical Analysis

For the purpose of statistical analyses, the data were weighted by the type of the secondary school, year of study, and by the administrative district of the region based on the regional high school register and the district public administration division of the region. In this paper, the results are presented after the application of the weights. Binary Logistic Regression models were used to estimate the odds of falling into the categories of risk of addictive behavior on the internet and gaming and of risk of problematic gambling. We also applied the same model to data on alcohol and/or cannabis use in order to point out different influences between behavioral and substance addictions. Altogether, we created five models for each risky behavior outcome variable (mEIUS, Lie/bet, CAGE, CAST, daily smoking), each of them with all abovementioned SES variables (as explanatory variables). Variables related to the characteristics of respondents, their study characteristics and parenting styles were included into the analysis to control the results. The data were processed using the IBM SPSS version 23 statistical software (IBM SPSS Inc., Chicago, IL, USA). The level of statistical significance for all analyses was set at α = 0.05 using two-tailed tests.

### 2.6. Ethical Approval

The study was reviewed by the Ethics Committee of the National Monitoring Centre for Drugs and Addictions under the reference number EKNMS-004/2019.

## 3. Results

### 3.1. Prevalence of Risky Behavior, SES, and Other Characteristics

For prevalence details about risky behavior, SES, and other characteristics, see Table 1.

The first row in Table 1 represents the total number of respondents showing signs of observed risky behavior. Out of the 1047 students, 11% (117) were at risk of excessive internet use or excessive gaming, 5% (53) were at risk of problematic gambling, 28% (293) were at risk of problematic alcohol use, 11% (113) were at risk of problematic cannabis use, and 12% (130) were smoking daily. The last column in Table 1 shows the frequency of the category in the whole sample, when the relative frequency of respondents at risk in the given category is calculated based on it. In the study sample, 53% were males. The average age was 17.49 years (male 17.53, female 17.44). Most students (51%) were studying at vocational schools with the state school-leaving exam (generally four years of study), 28% at vocational schools without the state school-leaving exam (three years of study), and 20% at general education schools (elite education). The same proportion of mothers and fathers (16% each) had a university degree. However, there was a higher proportion of mothers that finished secondary education (46% vs. 36% in fathers). Most students assessed their family status as average (62%), and only 9% of students as low. Twenty-nine percent of students reported they had more own money than their peers, and 29% of them thought they had less own money than their peers. Seventy-three percent of students reported they had moved once at most; 69% of respondents reported they lived in a two-parent family.

### 3.2. The Relationship between SES and Aehavioral Addictions

Table 2 describes two binary logistic models for each of the dependent variables (the risk of addictive behavior on the internet and gaming, and the risk of problematic gambling). There were statistically significant differences between the groups at-risk and not-at-risk for addictive behavior on the internet and gaming, while there were no significant differences for problematic gambling.

The stability of the family background as well as the subjective assessment of SES were not significant in either model. However, when calculated at a level of significance 0.10, the students who assessed their family status as higher had higher odds of problematic gambling than students who assessed their family status as average. The education of mothers did not play any significant role. Students whose fathers had university education engaged more likely in risky behavior in comparison with students whose fathers had secondary education with the state school-leaving exam. The students whose fathers had secondary education without the state school-leaving exam were at a higher risk in comparison with those whose fathers had secondary education with the state school-leaving exam.

No statistical differences were found in gender for the risk of addictive behavior on the internet and gaming. However, gender played a significant role in the risk of problematic gambling, lowering the odds for female students (0.04). The year of study was associated with both dependent variables. Students in the fourth year showed lower odds of falling into the category of the risk of addictive behavior on the internet and gaming in comparison with students in the first year (0.34). In contrast to that, students in the fourth year had higher odds of falling into the category of the risk of problematic gambling in comparison with students in first year (4.29). The odds of the risk of problematic gambling increased significantly in students who repeated a year of study (2.77).

Regarding parenting styles, significant differences were found only in the risk of addictive behavior on the internet or gaming between the authoritarian and authoritative styles. The models showed significant group differences between students engaged in risky activities with parents and students who did not. Students who reported gambling with parents showed higher odds of being at risk of addictive behavior on the internet or gaming (5.79) and problematic gambling (8.79). Furthermore, students who reported tobacco smoking with parents had lower odds of being at risk of addictive behavior on the internet or gaming (0.22). The students who reported using cannabis with parents had lower odds of falling into the category of problematic gambling (0.06).

### 3.3. The Relationship between SES and Substance Addictions

Three binary logistic models for each of the dependent variables (at risk of problematic use of alcohol, at risk of problematic use of cannabis, at risk of daily smoking) are presented in Table 2. Particular dimensions of SES showed significant effects on substance addictions in all models. The stability of the family background was not significant. Regarding the education-based SES, the education of mothers did not play any significant role. Only in the case of daily smoking, the fathers´ education was significant. Students whose fathers had university education were less likely to engage in daily smoking in comparison with those whose fathers had secondary education (0.31). The subjective assessment of SES was significant only if students assessed their own financial resources, while the family status was not significant in our models. Students that assessed their own financial resources as above average showed higher odds of being at risk of problematic use of alcohol (1.66) and at risk of problematic use of cannabis (2.85).

Regarding gender, no statistical differences were found in the risk of problematic use of alcohol and daily smoking. However, female students had lower odds of falling into the category of the risk of problematic use of cannabis (0.42). The odds of falling into the category of the risk of problematic cannabis use increased significantly in students who repeated a year of study (2.82). The models showed significant differences between the group of students that engaged in risky activities with parents, and the group that did not. Students that reported drinking alcohol with parents showed higher odds of being at risk of problematic alcohol use (2.12). Higher odds of being at risk of problematic cannabis use was found in students who reported smoking tobacco with parents (4.68) and/or smoking cannabis with parents (8.05). Odds of being at-risk of daily smoking was high in students who reported smoking tobacco with parents (28.88), while it was lower for those who reported gaming online and internet use with parents (0.51).

### 3.4. A Comparisons of Substance Addictions and Behavioral Addictions in Relation to SES

**Figure 1:**
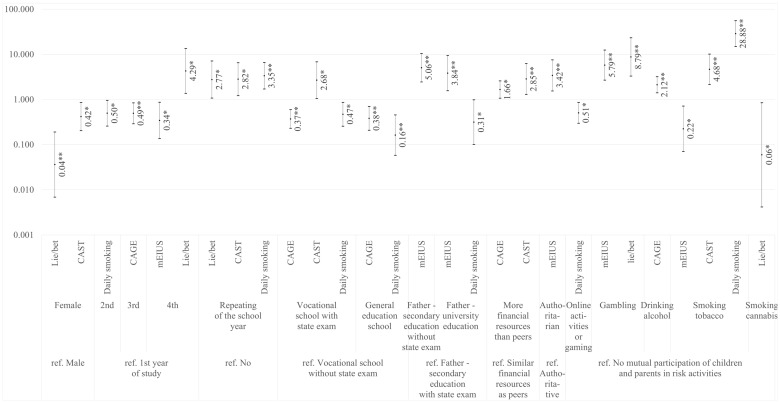
Odds ratios with 95% CI in multinomial regression models of mEIUS, Lie/bet, CAST, CAGE, and daily smoking for the statistically significant relationships; * statistically significant at 0.05 level; ** statistically significant at 0.01 level.

We used the same models to study the relationship between SES and substance addictions, and between SES and behavioral addictions (Figure 1). The education-based SES was significant in the case of excessive internet use risk, excessive gaming risk, and daily smoking. It was not significant in the case of the risks of problematic gambling, alcohol use and cannabis use. The subjective assessment of SES influenced only the risk of substance addictions, except for daily smoking. The students who reported above-average financial resources showed increased odds of problematic alcohol use risk and of cannabis use risk. Regarding control variables, we found that gender differences were significant only for the risk of problematic gambling and the risk of problematic cannabis use. In our models, the following relationships proved to be statistically significant: engagement with parents in behavioral addictions increased the odds of behavioral addiction risks and decreased the odds of substance addiction risks. Engagement with parents in substance addictions decreased the odds of behavioral addiction risks and increased the odds of substance addiction risks.

## 4. Discussion

Based on data from a structurally disadvantaged region in Central Europe, the Ústí nad Labem Region, this study found evidence for the relationship between SES and risks of some behavioral as well as substance addictions. At a significance level α = 0.05, we proved the relationship between SES and the risks of addictive behavior on the internet and gaming and/or substance use, and no statistically significant relationship between SES and risky problematic gambling. In this section, we will focus in greater detail on the relationships between SES and different forms of risky behavior in the following order: the risk of addictive behavior on the internet and gaming, the risk of problematic gambling, and the relationship between substance addictions and behavioral addictions. The section will be closed with limitations and strengths of the study.

Regarding the risk of addictive behavior on the internet and gaming, our findings were in line with Durkee et al. [20], Ko et al. [21] and Lee and McKenzie [5], the latter of which found a relationship between SES and the EIUS tool, while our findings were not in line with Blinka et al. [18] and Spilková et al. [19]. The differences between already-published findings and our results might be attributed to different populations studied. Blinka et al. [18] used the same measures when conducting his research in the general population of all European adolescents, and Spilková et al. [19] focused on the whole population of the Czech Republic. In our study, we focused on a specific subpopulation of a structurally disadvantaged region. However, this hypothesis requires further research. The generalizability of our findings might be lower; however, our findings are more specific, providing better applicability and a higher local relevance for preventive measures [39]

Focusing on the specific dimension of SES, fathers´ education had a significant effect on the risk of addictive behavior on the internet and gaming. Our research showed that both low and high status increased the chances of risky behavior as compared to the average status. Students whose father had either lower education or higher education than average had a higher chance of being at-risk. This is in line with findings of Humensky [40] on substance use, who found that high SES was also associated with higher rates of binge drinking, marijuana, and cocaine use. The effect of a family’s social economic status self-assessment manifested itself only partially. The stability of SES did not prove either for family structure or for moving. Our finding that living in a single parent family had no significant effect was in line with Blinka et al. [18] and Spilková et al. [19], while in disagreement with results of Durkee et al. [20]. We suppose that the effect of moving did not manifested itself, because our sample did not include students from socially excluded localities that are characterized by a high frequency of moving [41]. The effect of parental styles, mentioned for example by Kalmus et al. [42], proved only partially significant in our research. It was only in the case of the risk of addictive behavior on the internet and gaming where the authoritarian style increased the chances of being in the risk zone as compared to the authoritative style.

In the case of the risk of addictive behavior on the internet and gaming, the influence of the father’s education manifested itself strongly. This finding is consistent with the fact that the socioeconomic status is still determined gender-wise in the Czech Republic, where, e.g., the gender pay gap has been above the European average for a long time [43]. There are also differences in the fields of study in which men and women are educated. In terms of education, most women completed education in fields such as education and training, health and social care, or the humanities, while men were involved in technical sciences, construction manufacturing, science, mathematics, or physics [43].

The relationship between the risk of problematic gambling and SES and family characteristics did not manifest as strongly as in the case of the risk of addictive behavior on the internet and gaming. Like Molinaro et al. [7], we did not find the relationship between parental education, family structure, and gambling, which, on the other hand, appears in a later Molinaro et al. [10] study. However, shifting the value of the statistical significance to 10%, the subjective assessment of the family status appears significant. This must be interpreted with caution and investigated in future studies, but it suggests that students that rated their families as wealthier had a higher chance of an increased risk. This is partly in line with Molinaro et al. [10], who reported a reduction in the chances of problematic gambling for students with average SES as compared to the ones with higher SES. Molinaro et al. [10] reported on the effect of parental supervision, while we found no such influence in our study. The relationships between SES and substance addictions and SES and behavioral addictions differ. The relationship between education-based SES and the risk of addictive behavior on the internet and gaming and/or daily smoking was significant in contrast to the risk of problematic gambling, drinking, and cannabis use. In the case of the risk of addictive behavior on the internet and gaming, higher and/or lower parental education was riskier than their average education. As for daily smoking, a higher parental education lowered the risk. This suggests that risky behaviors such as alcohol drinking or gambling are prevalent in the whole population in the region under study, while daily smoking and the risk of addictive behavior on the internet and gaming are affected by the parental education. The subjective assessment of money available for personal needs was associated with the risk of substance addictions, except for daily smoking. Students reporting above-average financial resources showed increased odds of the risk of problematic alcohol use and/or cannabis use. It suggests that these kinds of risky behaviors require sufficient financial resources.

### Strengths and Limitations

In contrast to previous studies focusing on nation-wide [19] or all-European [10,18] populations of adolescents, we focused on adolescents in a structurally disadvantaged region. According to our knowledge, this is the first study to investigate the relationship between SES and the risk of behavioral addictions in a structurally disadvantaged region among student population. The choice of this area allowed us to monitor the interactions of SES with the rates of risky internet use, gambling, and addictive substance use in a particular subpopulation. However, this may also be considered to be a limitation of this study, since the results are not generalizable for the whole population, but only for the population of structurally disadvantaged regions. Since we have modified the EIUS tool [18] by extending its focus on the gaming behavior, we were able to identify a wider range of phenomena using a single tool; however, this tool was not standardized in terms of reliability and validity. Thus, the use of this scale could be seen as a limitation of the study as well as its strength. Another limitation is the possible selection bias, since the participation of schools was on a voluntary basis. However, we tried to compensate for this limitation by weighting data according to the type of school, year of study, and school district. A questionnaire survey based on self-assessment of students does not provide objective data about problematic behavior. On the other hand, the use of comparable tests for screening can be seen as a strength of our research.

## 5. Conclusions

The results highlight specific relations between SES and the risks of addictive behavior on the internet and gaming in structurally disadvantaged regions. We found that the risk of addictive behavior is not only related to the lower SES, but that it is raised both in students with lower SES and those with higher SES. We found no relationship between SES and gambling. In contrast to national and international studies, structurally disadvantaged regions may provide different results that will better inform prevention activities targeting specifically on the right groups at risk. To study the influence of SES in different countries and regions in detail, the significant differences in what constitutes SES in these regions need to be described and used in the analyses.

## Figures and Tables

**Table 1:** Prevalence of risky behavior—mEIUS, Lie/bet, CAGE, CAST, and daily smoking.

		mEIUS		Lie/bet		CAGE		CAST		Daily Smoking		Total	
Variable	Category	*n*	% in Category	n	% in Category	*n*	% in Category	*n*	% in Category	*n*	% in Category	*N*	% in Sample
Total sample		117	0.11	53	0.05	293	0.28	113	0.11	217	0.21	1047	1
Gender	Male	56	0.11	50	0.09	159	0.30	68	0.13	132	0.24	553	0.53
	Female	61	0.12	3	0.01	134	0.28	45	0.10	85	0.17	493	0.47
Year of study	First	36	0.11	12	0.04	101	0.32	35	0.11	72	0.22	331	0.32
	Second	41	0.15	12	0.04	82	0.29	38	0.13	49	0.17	286	0.27
	Third	31	0.14	15	0.06	69	0.27	26	0.11	54	0.22	259	0.25
	Fourth	8	0.05	14	0.08	41	0.24	15	0.09	41	0.24	171	0.16
Type of school	Vocational school without state exam	29	0.11	19	0.07	115	0.40	41	0.14	106	0.37	296	0.28
	Vocational school with state exam	67	0.13	22	0.04	124	0.24	62	0.12	100	0.19	538	0.51
	General education school	21	0.10	12	0.06	54	0.26	10	0.05	10	0.05	212	0.20
Repeating of the school year	Not once	95	0.11	40	0.04	259	0.29	89	0.10	166	0.19	912	0.87
	At least once	21	0.19	14	0.10	34	0.27	25	0.19	50	0.38	133	0.13
Expulsion from school	Not once	116	0.12	53	0.05	282	0.28	109	0.11	211	0.21	1030	0.99
	At least once	1	0.06	0	0	9	0.60	4	0.28	5	0.38	14	0.01
Parenting styles	Authoritative	19	0.06	13	0.04	70	0.23	17	0.06	40	0.13	306	0.31
	Authoritarian	31	0.20	4	0.02	41	0.26	17	0.11	17	0.11	154	0.16
	Permissive	31	0.13	13	0.06	70	0.30	33	0.14	75	0.32	234	0.24
	Neglecting	36	0.13	18	0.07	94	0.34	30	0.11	76	0.27	285	0.29
Online activities or gaming	No	40	0.10	20	0.05	106	0.26	30	0.08	91	0.23	409	0.42
	Yes	77	0.13	28	0.05	172	0.31	72	0.13	120	0.21	576	0.58
Gambling	No	90	0.10	30	0.03	237	0.27	77	0.09	172	0.20	883	0.90
	Yes	27	0.26	19	0.18	41	0.40	25	0.25	38	0.38	102	0.10
Drinking alcohol	No	32	0.09	16	0.04	73	0.21	20	0.06	61	0.17	357	0.36
	Yes	85	0.14	32	0.05	204	0.33	81	0.13	147	0.24	623	0.64
Smoking	No	100	0.13	35	0.04	200	0.26	46	0.06	78	0.10	778	0.79
	Yes	16	0.08	14	0.07	79	0.39	54	0.27	130	0.65	203	0.21
Cannabis smoking	No	103	0.12	47	0.05	240	0.27	60	0.07	168	0.19	899	0.92
	Yes	10	0.13	2	0.02	35	0.47	34	0.45	30	0.42	74	0.08
Use of other drugs	No	110	0.12	47	0.05	264	0.28	86	0.09	193	0.21	942	0.96
	Yes	7	0.19	1	0.04	15	0.41	16	0.45	17	0.50	36	0.04
Education of Father	Secondary with state exam	13	0.04	14	0.04	79	0.25	28	0.09	60	0.18	331	0.36
	Secondary without state exam	51	0.12	24	0.06	141	0.33	58	0.13	124	0.29	438	0.48
	University	22	0.16	11	0.08	43	0.31	9	0.07	11	0.08	141	0.16
Education of Mother	Secondary with state exam	50	0.12	22	0.05	129	0.30	48	0.11	81	0.19	438	0.46
	Secondary without state exam	41	0.12	20	0.06	91	0.26	48	0.14	98	0.28	360	0.38
	University	16	0.11	9	0.06	47	0.31	13	0.09	17	0.11	156	0.16
Subjective assessment of students’ own financial resources	Similar as peers	54	0.13	13	0.03	101	0.24	39	0.09	84	0.20	430	0.41
	Less than peers	42	0.15	15	0.05	99	0.33	33	0.11	66	0.22	305	0.29
	More than peers	21	0.08	25	0.08	92	0.31	41	0.14	67	0.23	303	0.29
Subjective assessment of family financial status	Average	81	0.13	21	0.03	182	0.29	71	0.11	138	0.22	641	0.62
	Poorer	8	0.09	6	0.07	22	0.25	11	0.12	19	0.22	90	0.09
	Richer	27	0.10	25	0.09	86	0.29	31	0.11	59	0.20	295	0.29
Frequency of moving	Once or never	96	0.13	39	0.05	197	0.27	76	0.10	143	0.19	764	0.73
	Twice or more	21	0.08	14	0.05	96	0.34	38	0.14	74	0.27	281	0.27
Family composition	One-parent family	46	0.15	12	0.04	92	0.29	47	0.15	55	0.18	324	0.31
	Two-parent family	71	0.10	41	0.06	201	0.28	66	0.09	162	0.23	723	0.69

Note: The Modified Excessive Internet Use scale (mEIUS), CAGE (acronym of the key words: cut, angry, guilty and eye-opener), Cannabis Abuse Screening Test (CAST).

**Table 2:** Multinomial logistic regression predicting likelihood of problem behavior—mEIUS, Lie-Bet, CAGE, CAST, and daily smoking.

		mEIUS			Lie-Bet			CAGE			CAST			Daily Smoking		
Variable	Parameter	OR	95% CI	*p*	OR	95% CI	*p*	OR	95% CI	*p*	OR	95% CI	*p*	OR	95% CI	*p*
Gender (ref. Male)	Female	10.551	0.867–2.776	0.139	**0.036**	**0.007–0.191**	**<0.001**	1.232	0.852–1.782	0.267	**0.419**	**0.204–0.861**	**0.018**	0.807	0.473–1.377	0.432
Year of study (ref. First)	Second	0.902	0.478–1.702	0.750	2.081	0.693–6.246	0.191	0.651	0.417–1.015	0.058	0.723	0.334–1.563	0.410	**0.497**	**0.258–0.956**	**0.036**
	Third	0.701	0.307–1.601	0.399	3.302	0.949–11.493	**0.061**	**0.494**	**0.290–0.842**	**0.010**	0.450	0.147–1.375	0.161	0.865	0.411–1.824	0.704
	Fourth	**0.344**	**0.136–0.871**	**0.024**	**4.294**	**1.365–13.505**	**0.013**	0.662	0.394–1.112	0.119	0.562	0.227–1.393	0.214	1.334	0.671–2.651	0.411
Type of school (ref. Vocational school without state exam)	Vocational school with state exam	1.277	0.608–2.685	0.518	0.954	0.339–2.684	0.929	**0.371**	**0.231–0.596**	**<0.001**	**2.684**	**1.055–6.832**	**0.038**	**0.471**	**0.257–0.865**	**0.015**
	General education school	1.525	0.582–3.994	0.390	0.850	0.232–3.114	0.806	**0.382**	**0.209–0.699**	**0.002**	1.554	0.404–5.970	0.521	**0.163**	**0.058–0.455**	**<0.001**
Repeating of the school year (ref. No)	Yes	2.015	0.872–4.657	0.101	**2.768**	**1.075–7.129**	**0.035**	1.265	0.706–2.265	0.429	**2.824**	**1.219–6.541**	**0.015**	**3.355**	**1.708–6.590**	**<0.001**
Expulsion from school (ref. No)	Yes	0.000	0.000–0.000	0.999	0.000	0.000–0.000	0.999	6.344	0.866–46.478	0.069	0.000	0.000–0.000	0.999	1.137	0.121–1.651	0.910
Parenting styles (ref. Authoritative)	Authoritarian	**3.416**	**1.546–7.547**	**0.002**	0.305	0.075–1.235	0.096	1.135	0.658–1.956	0.649	0.432	0.144–1.296	0.134	0.414	0.168–1.019	0.055
	Permissive	1.796	0.827–3.899	0.139	0.433	0.144–1.304	0.137	1.251	0.776–2.015	0.358	0.844	0.361–1.971	0.695	1.466	0.736–2.917	0.276
	Neglecting	1.309	0.599–2.862	0.500	0.510	0.193–1.346	0.174	1.330	0.833–2.124	0.232	0.752	0.317–1.787	0.519	1.702	0.914–3.170	0.094
Mutual participation of children and parents in (ref. No)	Online activities or gaming	1.320	0.753–2.313	0.333	1.105	0.493–2.476	0.809	1.307	0.908–1.882	0.150	0.889	0.442–1.787	0.740	**0.506**	**0.298–0.860**	**0.012**
	Gambling	**5.793**	**2.687–12.488**	**<0.001**	**8.793**	**3.309–23.364**	**<0.001**	1.311	0.741–2.321	0.352	1.645	0.669–4.047	0.278	1.656	0.770–3.561	0.197
	Drinking alcohol	0.745	0.416–1.334	0.323	0.904	0.379–2.153	0.819	**2.123**	**1.407–3.205**	**<0.001**	1.255	0.570–2.761	0.573	0.575	0.327–1.010	0.054
	Smoking	**0.225**	**0.071–0.714**	**0.011**	1.814	0.630–5.227	0.270	1.225	0.761–1.973	0.403	**4.676**	**2.152–1.161**	**<0.001**	**28.878**	**14.928–55.864**	**<0.001**
	Cannabis smoking	0.684	0.156–2.994	0.614	**0.060**	**0.004–0.847**	**0.037**	0.933	0.441–1.971	0.855	**8.053**	**3.310–19.591**	**<0.001**	0.636	0.242–1.670	0.358
	Use of other drugs	2.818	0.458–17.346	0.264	1.297	0.082–2.563	0.854	1.063	0.322–3.506	0.921	0.381	0.091–1.599	0.187	0.275	0.061–1.241	0.093
Education of Father (ref. Secondary education with state exam)	Secondary education without state exam	**5.064**	**2.451–1.464**	**<0.001**	1.415	0.545–3.675	0.476	0.937	0.628–1.400	0.753	1.600	0.789–3.246	0.193	1.220	0.702–2.118	0.481
	University education	**3.839**	**1.560–9.446**	**0.003**	1.775	0.520–6.060	0.360	1.088	0.617–1.917	0.770	0.679	0.189–2.436	0.552	**0.315**	**0.101–0.982**	**0.046**
Education of Mother (ref. Secondary education with state exam)	Secondary education without state exam	0.777	0.390–1.548	0.473	1.391	0.537–3.604	0.497	0.792	0.520–1.206	0.277	**0.475**	**0.225–1.004**	**0.051**	1.292	0.749–2.226	0.357
	University education	1.454	0.674–3.136	0.340	0.617	0.175–2.173	0.453	1.490	0.891–2.491	0.128	0.861	0.321–2.311	0.766	0.823	0.345–1.960	0.659
Subjective assessment of students’ own financial resources (ref. Similar)	Less financial resources than peers	1.013	0.527–1.947	0.969	2.310	0.805–6.634	0.120	1.462	0.941–2.271	0.091	1.501	0.645–3.491	0.346	1.261	0.672–2.365	0.471
	More financial resources than peers	0.592	0.297–1.178	0.135	2.216	0.842–5.832	0.107	1.663	1.072–2.582	0.023	**2.852**	**1.302–6.245**	**0.009**	1.560	0.855–2.846	0.147
Subjective assessment of family financial status (ref. Average)	Poorer	0.904	0.334–2.449	0.843	0.988	0.244–3.992	0.986	0.562	0.291–1.086	0.087	0.380	0.098–1.478	0.162	0.454	0.187–1.104	0.081
	Richer	1.113	0.576–2.151	0.750	2.345	0.933–5.894	0.070	0.735	0.475–1.139	0.169	0.702	0.327–1.506	0.364	1.323	0.726–2.412	0.360
Frequency of moving (ref. Once or never)	Twice or more	0.511	0.249–1.050	0.068	0.723	0.271–1.926	0.517	1.358	0.913–2.020	0.131	0.958	0.465–1.975	0.908	1.479	0.861–2.540	0.156
Family composition (ref. One-parent family)	Two-parent family	0.954	0.503–1.808	0.886	0.887	0.326–2.416	0.815	1.060	0.710–1.580	0.776	0.580	0.280–1.201	0.142	1.192	0.680–2.090	0.540

Note: Statistically significant coefficients are in bold, The Modified Excessive Internet Use scale (mEIUS), CAGE (acronym of the key words: cut, angry, guilty and eye-opener), Cannabis Abuse Screening Test (CAST).
